# Comparative intrafollicular and plasma iron, ferritin, and transferrin concentrations in cycling mares

**DOI:** 10.14202/vetworld.2024.2370-2375

**Published:** 2024-10-27

**Authors:** Katiuska Satué, Esterina Fazio, Gemma Velasco-Martinez, Cristina Cravana, Deborah La Fauci, Pietro Medica

**Affiliations:** 1 Department of Animal Medicine and Surgery, Faculty of Veterinary Medicine, CEU-Cardenal Herrera University, Tirant lo Blanc, 7, Alfara del Patriarca, 46115 Valencia, Spain; 2 Department of Veterinary Sciences, Veterinary Physiology Unit, University of Messina Polo Universitario Annunziata, Via Palatucci 13, 98168 Messina, Italy

**Keywords:** ferritin, follicular fluid, iron, mare, transferrin

## Abstract

**Background and Aim:**

In females of various species and experimental animals, iron (Fe) status in follicular fluid (FF) is associated with local physiological reproductive events related to follicle development, steroidogenesis, and oocyte maturation. However, these mechanisms remain unknown. This study aimed to determine and compare the intrafollicular and plasma concentrations of Fe, ferritin (Ferr), and transferrin (TRF) in cycling mares.

**Materials and Methods:**

Sixty ovaries were collected during the breeding season from 30 clinically normal mares raised for slaughterhouse meat production. Blood samples were collected before slaughter. Follicles were classified into three categories according to size: Small (20–30 mm; n = 20), medium (≥31–40 mm; n = 20), and large (≥41 mm; n = 20). The FF samples, after collection, were immediately taken to the laboratory for processing and were centrifuged, and the Fe and Ferr concentrations in the supernatant and plasma were determined by spectrophotometry.

**Results:**

Although intrafollicular Fe and Ferr were similar to plasma, TRF was significantly higher in FF than in systemic circulation (p < 0.05). Follicular development does not modify the status of Fe in the mare.

**Conclusions:**

Based on this evidence, it is possible that the acquisition of this molecule possibly originated from a local *de novo* source, whereas their diffusion through ultrafiltration does not play a relevant role. These results provide new scientific insights into the status of follicle Fe, suggesting its involvement in normal ovarian functions in mares.

## Introduction

Iron (Fe) metabolism at the cellular and systemic levels is tightly regulated to maintain homeostasis. Fe is an essential element for cells that perform biological functions associated with the transport and storage of oxygen, synthesis of hormones, mitochondrial energy metabolism, generation of ATP, mediation of oxidation-reduction reactions, and synthesis and repair of DNA [1, 2]. Ferritin (Ferr) captures and buffers free Fe at the intracellular level, making this molecule available for cellular processes and protecting lipids, DNA, and proteins from the potentially toxic effects induced by Fe [3]. Transferrin (TRF) is a β-globulin produced mainly by the liver and is responsible for transporting Fe to storage tissues [4].

At the reproductive level, Fe participates in various processes, including estrous cycle regularity, ovarian steroidogenesis, follicular development, and ovulation, as it is a cofactor of several metabolic enzymes [5]. Although most intrafollicular TRFs originate from the peripheral circulation, the small part is produced by granulosa cells (GCs) in humans [6, 7], mice [8, 9], and sows [10]. TRFs of plasma origin enter the follicle through endocytosis by GCs through a soluble transporter that captures Fe released in the interstitial space [11, 12]. The TRF receptor, which has a high affinity for TRF, is essential for the internalization of the TRF-Fe complex into cells, where Fe is released into acidic lysosomes for use by GCs [6]. As the follicle develops, TRF receptors actively proliferate in GCs and increase the concentrations of TRF in follicular fluids (FFs) [10, 13, 14], supporting the hypothesis that TRF in FFs results mainly from local synthesis by CGs.

Previous studies [6, 15–17] in women have shown a close relationship between the rate of follicular maturation, steroidogenesis, and follicular TRF and its receptors in these cells. The involvement of TRFs in steroidogenesis and oocyte maturation can be explained based on evidence in sows by Tonai *et al*. [10]. These researchers demonstrated that preculture of porcine cumulus cells with follicle-stimulating hormone and TRF improved estradiol production, cumulus cell proliferation, and meiotic maturation of oocytes, resulting in increased oocyte competence. According to this study and the aforementioned findings, TRF in FF could be a potential biomarker for folliculogenesis and oocyte maturation.

Little is known about the intrafollicular Fe status at physiological levels, specifically in the mare. Most research has been conducted in human patients and experimental animals under pathological conditions that significantly alter the concentrations of Fe, Ferr, and TRF. Indeed, several studies in women with endometriosis, endometriomas, and polycystic ovary syndrome (PCOS) [12, 18–22] and in experimental animals, such as mice [23] and mice [24], concluded that higher Fe and Ferr levels and higher/lower TRF adversely affect the development and quality of oocytes *in vitro*, promoting meiotic abnormalities, chromosomal instability, and infertility [24–27]. Therefore, mitigating the impact of Fe stress on the follicular microenvironment, such as using antioxidant agents or Fe chelators, is expected to be an effective approach for preventing and treating infertility in these conditions [22].

Although Fe concentrations were recently assessed in both pregnant [28–30] and cyclic Spanish Purebred mares [31], as well as in FF [32], the Ferr and TRF concentrations have not been documented nor have their FF relationship with plasma. Considering the clinical use of Fe and Fe metabolites in women and experimental animals for the diagnosis of infertility, the hypothesis was that the detection of Fe status in FF may provide a better understanding of intrafollicular signaling and can be used as a possible biomarker of oocyte health in mares undergoing *in vitro* fertilization (IVF).

Therefore, this study aimed to determine the concentrations of Fe, Ferr, and TRF in FF and blood samples, considering the correlations between these molecules and the possible contribution of plasma ferric status to the FF content.

## Materials and Methods

### Ethical approval

All methods and procedures used in this study were in compliance with the guidelines of Spanish law (RD 37/2014) that regulate the protection of animals at the time of slaughter and the EU directive (2010/63/EU) on the protection of animals used for scientific purposes. The experimental protocol required for the animal studies was approved by the Animal Experimentation Ethics Committee (CEEA) of the CEU-Cardenal Herrera University (No. CEEA 23/01).

### Study period and location

The study was conducted during the breeding season months of April and May 2018 in the northern hemisphere, as described by Satué *et al*. [29]. During this period, the ambient temperature ranged from 27°C–31°C, with a relative humidity of 40%–60%. The slaughter was localized in Valencia (Spain), with geographic coordinates of latitude: 39° 31′ 0.01″ N and longitude: 0° 25′ 0.01″ E.

### Animals

A total of 30 clinically healthy mares (local autochthonous mares for meat production, which mainly include Draft, Hispano–Breton, and related crosses), aged 6.6 ± 1.3 years, were studied. According to Henneke *et al*. [33], the animals had a body condition score of 7–8 out of 9, with a mean weight of 533 ± 7.3 kg. All animals were subjected to the same management and feeding conditions, including orchard grass–alfalfa mixed hay, and had free access to mineral salt and fresh water in a sheltered area. The official veterinarians for each stockyard and slaughterhouse accepted responsibility for the study participation, and only mares with a reproductive history of normal estrous cycles were included in this study. The veterinary examination of the animals before slaughter consisted of a careful review of official documentation, which included livestock of origin, sanitary registration number, suitable health status, deworming and vaccination plan, and the clinical and reproductive history of the animals, along with the clinical normal reproductive tracts after slaughter. The inclusion criteria for the animals were: (1) Absence of reproductive diseases in the clinical examination; (2) absence of inflammatory processes or infections that had required treatment or hospitalization during the month before the onset of the study; (3) vaccination and deworming correctly; (4) younger than 15 years old with no conformation defects that affect the perineum and vulva; and (5) normal involution of the uterus in previous births and lack of previous history of reproductive diseases that affect fertility.

### Blood and ovaries collection

Before slaughter, blood samples (20 mL) were collected from the jugular vein using 20 mL disposable syringes with a luer cone (Becton Dickinson Discardit^®^ II, Fraga, Spain) attached to 40 mm 18–20 G needles (Sterican^®^, Braun Melsungen AG, Melsungen, Germany) and transferred to heparinized tubes (Tapval^®^, Barcelona, Spain). Blood samples were centrifuged (J.P. Selecta^®^ centrifuge, Barcelona, Spain) at 3000× *g* for 10 min at 4°C, after 30 min, and the plasma obtained was stored at −20ºC until analysis.

In postmortem, the ovaries of all mares were collected; the time between slaughter and collection of the ovaries was <2 h, as reported by Hinrichs [34]. All ovaries were placed in containers containing 0.9% physiological saline plus penicillin (100 IU/mL) and streptomycin (50 mg/mL) and transported to the laboratory in individually labeled plastic bags in thermal containers (at 25°C) [35].

### FF collection

The ovaries were washed 3 times with sterile saline solution, and the follicles were directly measured with a digital Vernier caliper and categorized according to the diameter as small (20–30 mm; n = 20), medium (≥31–40 mm; n = 20), or large (≥41 mm; n = 20). Then, the FF was aspirated using different sterile syringes and needles of 22G for each follicle and centrifuged for 10 min at 1200× *g* to eliminate the cumulus-oocyte complexes; only the supernatant, represented as pure FF, was collected and stored in aliquots of 0.5 mL at −20°C until analysis.

### Determination of Fe, Ferr, and TRF concentrations in plasma and FF

Serum and intrafollicular Fe (μg/dL), Ferr (μg/dL), and TRF (mg/dL) concentrations were analyzed by a Spin 200E spectrophotometer (Spinreact®, Barcelona, Spain) using commercial house reagents based on colorimetry for Fe (FerroZine) and turbidimetry for Ferr (Latex) and TRF (Spinreact^®^, Barcelona, Spain). The sample detection limits for Fe, Ferr, and TRF were 0.850 μg/dL to linearity limits of 1000 μg/dL, 5.04 μg/dL, and 94 mg/dL, respectively. The intra and inter-assay coefficients of variation were 0.79 % and 3.17 %, to 5.1 % and 6.3 %, and 1.7% and 2.1% for Fe, Ferr, and TRF, respectively.

### Statistical analysis

Descriptive statistics mean ± standard deviation (SD) for Fe, Ferr, and TRF concentrations in FF of small, medium, and large follicles and in blood plasma were calculated. Normality was verified in all data groups using the Kolmogorov–Smirnov test. To determine the magnitude of variation in the concentrations of plasma and FF constituents, data were subjected to a one-way analysis of variance. The relationship between FF and the systemic Fe, Ferr, and TRF concentrations was examined by linear regression analysis, and the correlation was expressed using Pearson’s correlation coefficient. Differences were considered statistically significant when p < 0.05.

## Results

Table-1 expresses the descriptive statistics, including the mean, SD, and maximum and minimum values in plasma and FF of small, medium, and large follicles.

**Table-1:** Concentrations (mean ± standard deviation) and the maximum and minimum values of Fe, Ferr and TRF both in plasma and in the FF of different follicular sizes.

Parameters	Plasma		FF			
	
	No.	Mean	No.	Small (20-30 mm)	Medium (≥ 31-40 mm)	Large (≥ 41 mm)
Fe (µg/dL)	76	180.7±50.6 (155.0–206.5)	20	165.0±26.1 (148.9–180.4)	157.1±23.5 (140.3–173.9)	168.3.6±25.3 (148.8–187.8)
Ferr (µg/dL)	76	162.5±16.0 (154.2–170.7)	20	156.6±6.62 (152.6–160.6)	149.6±7.91 (143.9–155.2)	149.7±4.71 (146.1–153,4)
TRF (mg/dL)	76	262.9±17.2 (254.1–271.8)	20	338.3±35.1 (317.1–359.5)	307.9±20.1 (293.5–322.2)	336.3±36.5 (308.2–364.4)

Fe=Iron, Ferr=Ferritin, TRF=Transferrin, FF=Follicular fluid

**Figure-1:**
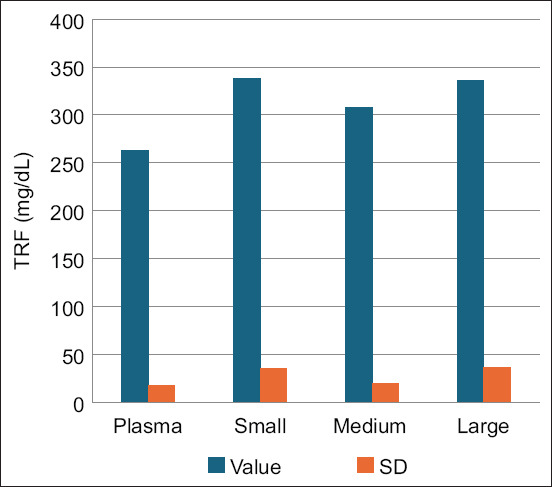
Concentrations of transferrin (TRF) in plasma and follicular fluid from different follicles of mares. Plasma versus small, medium, and large follicles: *p < 0.05.

Although Fe and Ferr did not differ significantly between plasma and FF, TRF increased in all FF samples of different follicular sizes compared with plasma (Figure-1). The correlation coefficients between plasma and intrafollicular concentrations of Fe, Ferr, and TRF are expressed in Table-2.

**Table-2:** Systemic and intrafollicular Fe, Ferr and TRF correlation coefficients in follicles of mares.

Parameters	Fe (µg/dL)	Ferr (µg/dL)	TRF (mg/dL)
Fe (µg/dL)	-	0.15	0.10
Ferr (µg/dL)	-	-	-0.22

Fe=Iron, Ferr=Ferritin, TRF=Transferrin, FF=Follicular fluid

None of the parameters in FF were correlated with plasma levels.

## Discussion

To the best of our knowledge, this study is the first to report parameters related to Fe status in FF and its comparison with plasma levels in mares.

In the mare, plasma and intrafollicular concentrations of Fe and Ferr were similar, although TRF was significantly higher in FF than in plasma. In a similar way to what occurs in the mare, no significant differences were observed for Ferr in the serum and FF compartments in women [16]; however, Ferr levels were slightly higher in the serum than in the FF compartment. Regarding the concentration of TRF, the results are contradictory; indeed, Mantzavinos *et al*. [16] reported that serum TRF increased in relation to those of FF for both pregnant and not-pregnant groups, which represented 36% and 43% of the corresponding serum levels, respectively. Angelucci *et al*. [13] and Jarkovska *et al*. [14] found significantly higher TRF in FF than plasma levels. In contrast to the data provided in this study, lower concentrations of Fe and TRF in porcine FF were collected from small and medium antral follicles than from large antral follicles [10]. However, it is possible to speculate that species differences and the different harvest times and cycle stages in which the FF samples were extracted influenced these differences. Previous studies in humans [6] and mice [8, 9] have shown that TRF originates mainly in the plasma, although *de novo* synthesis produces a small amount of TFR for GCs. TFR is absorbed by GCs through its soluble transporter [11, 12]. During maximum follicular growth, these TRF receptors actively proliferate in the GCs, so the uptake and intracellular distribution of Fe increase significantly [12]. This physiological mechanism causes a significant increase in TRF concentrations in FF, reaching significantly higher values than those in plasma [13, 14]. Aleshire *et al*. [6] showed that mature oocytes with FF TRF levels <220 mg/dL exhibit successful results in IVF. However, TRF concentration varies between different follicles in the same patient and may be 2–3 times that of serum [15]. Since none of the samples were bloody, the contribution of proteins derived from traumatic contamination with serum would be negligible and further diminished by FF dilution. Thus, contamination cannot account for the TRF concentrations observed in this study.

Given that follicular development expresses rapid cell proliferation and greater steroid generation, it would be logical to think that this process would imply a greater demand for Fe in mares, as occurs in porcine [10]. Some studies on FF in women [6, 15, 16] and in porcine [10] have shown that the rates of follicular maturation and the TRF, along with its receptors in these cells, are closely related. TRF concentrations in FF are highly correlated with circulating levels, the degree of follicular maturity, and steroidogenesis. In mares, the absence of correlations between intrafollicular and plasma TRF related to the increase of this molecule in FF could support the hypothesis that TRF in FF results mainly from local ovarian synthesis as GCs. However, the possibility of plasma contribution cannot be ignored. Our study did not investigate Ferr- and TRF-secreting cells in the FF, whose information would have provided a complete view of the situation; therefore, future studies are necessary to elucidate these aspects specifically.

The fact that Fe and Ferr levels are maintained during follicular development and that TRF levels increase could indicate that Fe is possibly used or bound to the binding protein in the FF of intact mares. Perhaps this mechanism could represent the preservation of the toxic effect that Fe exerts on the oocyte. Because the FF is the microenvironment for oocyte maturation and blastocyst formation, the abnormal microenvironment is affected by Fe overload, which induces impaired reproductive function [22]. Excess Fe alters redox homeostasis and leads to the formation of hydroxyl radicals. Hydroxyl molecules are highly toxic and, when formed, oxidize any nearby chemical groups capable of reacting, including DNA, lipids, and proteins, leading to cell death or DNA mutations and destruction of follicle GCs in the ovary [36]. Previous studies [12, 18–20, 37, 38] examined values of Fe, Ferr, and TRF in FF at the oocyte retrieval stage in fertile and sub-fertile women with endometriosis, endometriomas, polycystic ovary syndrome, and who underwent IVF. Significantly higher Fe and Ferr concentrations and lower TRF concentrations were observed in sub-fertile than fertile women, and the results of the IVF program were successful. More significant Fe overload in the infertile group negatively affects follicular development and the quality of oocytes *in vitro* due to the loss of antioxidant defenses [12, 20, 39].

In the same way, Fe plays a key role in the pathogenesis of endometriosis and infertility in a mouse model [23] and leads to a high degree of apoptosis and ferroptosis in murine embryos [24]. Indeed, Fe-overloaded FF can trigger ferroptosis in mouse GCs and the maturation of oocytes, thereby increasing the risk of endometriosis-related infertility [40]. The Fe-overloaded environment of FF not only inhibited the expression of glutathione peroxidase 4 and its upstream regulatory target glutathione but also increased the expression of nuclear receptor coactivator 4 (NCOA4) in GCs. This would lead to NCOA4-dependent ferritinophagy, increasing lipid peroxidation in GCs. Moreover, GCs undergoing ferroptosis cannot exert nutritional and paracrine functions on oocytes, and they can release GC exosomes containing abnormal microRNAs and inhibit murine oocyte maturation [41].

Although the addition of TRF to bind excess Fe reversed these effects, improving the ripening rate [42], high TRF levels are responsible for the formation of non-TRF-bound Fe, a toxic form of Fe with a propensity to induce oxidative stress (OS) [43]. OS can cause reactive oxygen species (ROS) at the FF level, which leads to lower embryonic quality by promoting meiotic abnormalities, chromosomal instability, and infertility in mice with endometriosis [43] and in humans with PCOS [25, 26]. The affected pathways included key biological processes, such as steroid metabolism, response to OS, and cell growth regulation, which could explain the reduction in oocyte quality [21, 27]. Therefore, mitigating the impact of Fe stress on the local microenvironment, such as using antioxidant agents or Fe chelators, is expected to be an effective approach for preventing and treating infertility in these conditions [22].

Since it is unknown to what extent specific reproductive pathological processes, such as endometritis, can affect the intrafollicular microenvironment, the determination of the levels of Fe, Ferr, and TRF in FF could represent a valuable tool in the detection of the molecular bases related to the loss of fertility in mares.

## Conclusion

For the first time, specific reference values for Fe, Ferr, and TRF concentrations according to follicle size and their relationship with plasma levels in physiologically normal mares have been established. However, future studies are necessary to evaluate the extent to which the Fe status influences follicular development and oocyte maturation in mares.

## Data Availability

The datasets generated and analyzed during the current study are available from the corresponding author upon reasonable request.

## Authors’ Contributions

KS, EF, and PM: Design and conception of the study. GVM: Conducted the study. DLF and CC: Data analyses. KS, EF, and CC: Manuscript preparation. All authors have read and approved the final manuscript
